# Estimating the severity distribution of disease in South Korea using EQ-5D-3L: a cross-sectional study

**DOI:** 10.1186/s12889-016-2904-5

**Published:** 2016-03-08

**Authors:** Minsu Ock, Min-Woo Jo, Young-hoon Gong, Hyeon-Jeong Lee, Jiho Lee, Chang Sun Sim

**Affiliations:** Department of Preventive Medicine, University of Ulsan College of Medicine, Seoul, South Korea; Department of Preventive Medicine, Korea University College of Medicine, Seoul, South Korea; Department of Occupational and Environmental Medicine, Ulsan University Hospital, University of Ulsan College of Medicine, 877 Bangeojinsunhwan-doro, Dong-gu, Ulsan, 682-714 South Korea

**Keywords:** Disease severity, Prevalence, EQ-5D

## Abstract

**Background:**

There is a paucity of data on the distribution of disease severity. In this study, we estimated disease severity distributions in South Korea using two EQ-5D-3L population surveys.

**Methods:**

A total of 110 health states for 35 diseases with 2–5 severity levels (e.g., mild, moderate, severe) were included in this study. A general population of 360 participants from the areas surrounding Seoul and Gyunggi evaluated these health states using EQ-5D-3L via face-to-face interviews and a paper questionnaire. The EQ-5D indices were used to measure the severity levels of health states and used as the cutoff points for the disease severity distributions. Finally, these cutoff points were applied to disease prevalence data with EQ-5D-3L, which were obtained from the Korean National Health and Nutrition Examination Surveys (KNHNES) and Korean Community Health Survey, in order to estimate the disease severity distributions.

**Results:**

The severity distributions of 8 diseases were estimated, including asthma, angina, stroke, chronic obstructive pulmonary disease, major depressive disorder, musculoskeletal problems in the legs, anemia, and allergic rhinitis and conjunctivitis. For example, the EQ-5D indices for chronic obstructive pulmonary disease severity were 0.929, 0.742, and 0.620, and the cut-off points were 0.835 (between mild and moderate) and 0.681 (between moderate and severe). Using these cutoff points, the distributions of chronic obstructive pulmonary disease severity were 66.5 % (mild), 23.3 % (moderate), and 10.1 % (severe) according to KNHNES.

**Conclusions:**

The estimated severity distributions in this study can be used as a valid calculation of the disease burden in the general population.

**Electronic supplementary material:**

The online version of this article (doi:10.1186/s12889-016-2904-5) contains supplementary material, which is available to authorized users.

## Background

The disability-adjusted life year (DALY) is a summary measure of overall disease burden and is expressed in terms of the number of years lost due to poor health, disability, or early death [1]. DALY has 2 components: years of life lost (YLLs) and years lived with disability (YLDs). This measure was first developed in 1990 as an approach for comparing the overall health and life expectancies of different countries [2]. Recently, the Global Burden of Disease (GBD) study group adopted a prevalence-based approach rather than an incidence-based approach [3]. Using both approaches, the YLL component is calculated using the same principle, which takes advantage of the number of deaths and standard life expectancy at age of death in years. However, when determining the YLD component, there are some differences between the 2 approaches in terms of disease duration, disability weight, and comorbidity [4]. Using the prevalence-based approach, disease duration is not directly considered and the disability weights are applied to the disease sequelae rather than the disease itself. In addition, it is easier to consider comorbidity using the prevalence-based approach than the incidence-based approach.

Notably, the prevalence-based approach uses big changes related to disability weight. In fact, after the development of DALYs, there have been some debates on the measurement of health loss, the use of person trade-offs, disability weights of whose perspectives, and the universality of disability weights [5]. In their 2010 study, the GBD group conducted international surveys on the general public using paired comparisons to estimate disability weights [6]. These changes make it easier to calculate DALYs, but this approach requires more data that were not needed when using the incidence-based approach, such as data about severity distribution [7]. Using the prevalence-based approach, the GBD group attempted to consider sequelae severity and briefly described the health state and sequelae severity [6]. In order to apply data on severity distributions and calculate DALYs, the GBD group asked a convenient sample of participants to evaluate SF-12v2 [8] for a hypothetical person, who was depicted as living with a certain health state from among 60 possible health states [9]. The GBD group then used population survey data from the United States and Australia to estimate marginal severity distributions.

They adapted this method because data on severity distributions are often scarce. However, applying the data on severity distributions from one country to another would impose limitations due to differences in race, economic factors, and healthcare system accessibility [10, 11]. In South Korea, two population surveys are available, the Korean National Health and Nutrition Examination Surveys (KNHNES) and Korean Community Health Survey (KCHS), which have prevalence data and health-related quality of life (HRQoL) data using EQ-5D-3L [12]. Therefore, disease severity distributions could be determined from the KNHNES and KCHS modifying the method used in the GBD study. In our current study, we estimated disease severity distributions in the Korean general population using two EQ-5D-3L population surveys and health state valuation survey data.

## Methods

### Study participants

A general population of 360 adults (≥19 years) from the areas surrounding Seoul and Gyunggi participated in this study. The study participants were recruited and stratified according to age, sex, and education using data from the 2010 Census of Korea. The sample size was determined by allocating 30 participants to each health state group (12 health state groups).

### Ethical considerations

This survey was conducted by a commercial survey company, who used face-to-face interviews and paper questionnaires after obtaining informed consent. This study was approved by the institutional review board of Asan Medical Center (S2014-1677-0002).

### Health state valuation survey procedure and health states

First, sociodemographic characteristics were determined, such as sex, age group, region, and education level. Second, each study participant described their own health states using EQ-5D-3L to adapt to the instrument. Lastly, the study participants were asked to complete EQ-5D-3L for 9 or 10 hypothetical people, as described by the lay descriptions of health states in the order of good health states.

In total, 110 health states of 35 diseases with 2–5 severity levels (e.g. mild, moderate, and severe) were included in this study. Those health states mainly originated from 220 health states, which were described in the 2010 GBD study [6]. Each health state was depicted in terms of the lay descriptions, which described the status of each health state in terms of several health aspects. Because the lay descriptions were originally developed in English, MO first translated these descriptions, which were rechecked by MWJ. In addition, 4 diseases—allergic rhinitis and conjunctivitis, annoyance, sleep disturbance, and cognitive impairment in children—were included in this study, because of local national burden of disease study for environmental diseases. The health states of additional 4 diseases were drafted by 2 authors (MO and MWJ) after referencing the existing lay descriptions reported by a previous study [6]. These 110 health states were divided into 12 groups, which were composed of 9–10 health states. Thirty participants were allocated to each health state group, therefore, each health state had 30 EQ-5D-3L responses. Exceptionally, the 3 health states related to anemia included 2 groups, so each health state of anemia had 60 EQ-5D-3L responses. We considered that at least 30 EQ-5D-3L responses using mean as a representative value would make parametric statistical tests possible. Table 2 lists the diseases and severity levels.

### Analysis

**Fig. 1:**
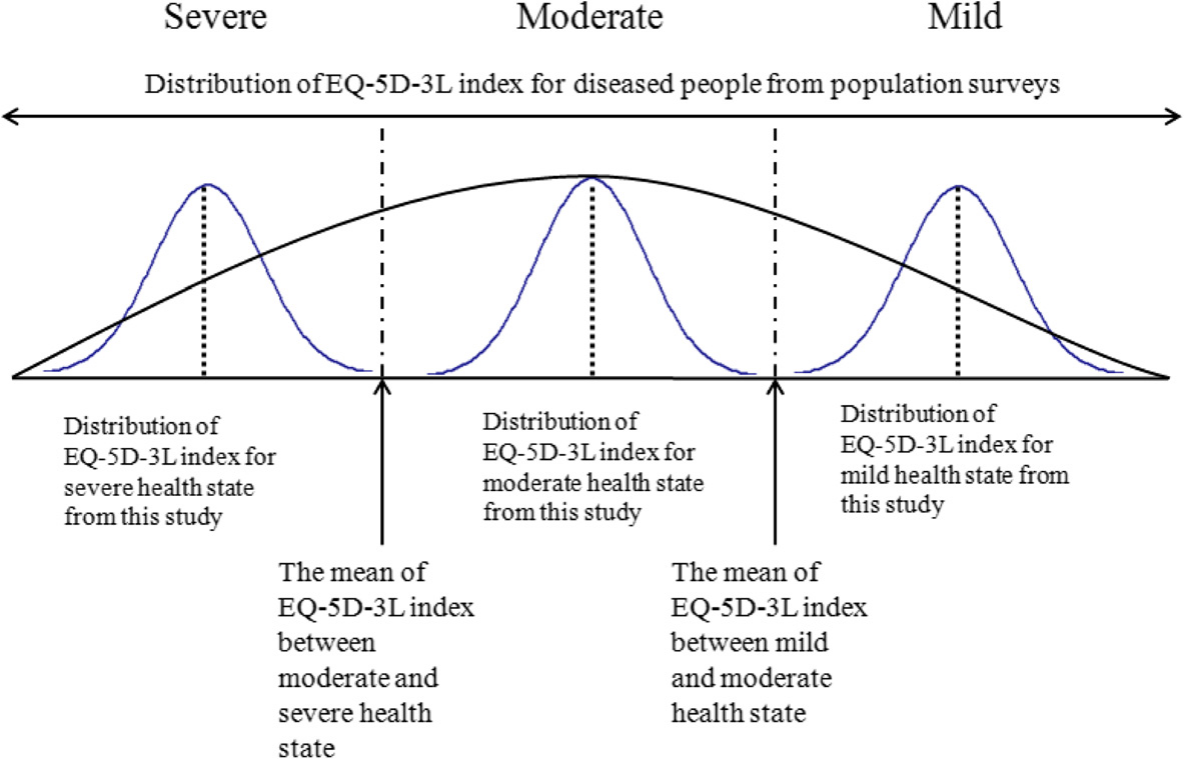
Approach used in this study to estimate disease severity distribution

Descriptive analyses of the basic characteristics of the study participants were first conducted. Then, the severity distributions were estimated using survey data obtained by this study and prior population survey data. Figure 1 shows the approach for estimating the severity distributions of the health states in this study. The EQ-5D-3L responses from each health state were transformed to the EQ-5D-3L index using the Korean EQ-5D-3L value set [13]. We used EQ-5D-3L rather than SF-12v2 because the KNHNES and KCHS adapted EQ-5D-3L to measure HRQoL. KNHNES and KCHS report different self-reported prevalence data by year. The cutoff points for the severity distributions of each disease were determined according to the averages of the mean values of the EQ-5D-3L index for the severity levels of the health states. Finally, these cutoff points were applied to the disease prevalence data from KNHNES and KCHS in order to estimate the disease severity distributions. We used pooled data from KNHNES (obtained between 2005 and 2012) and KCHS (2008–2012), respectively. All statistical analyses were conducted using SPSS 21.0 software.

## Results

The basic characteristics and self-perceived HRQoL values of the study participants are listed in Table 1. In total, 50.6 % of the study participants (182 participants) were female. Participants in their 40s and residents of Gyunggi were the largest groups. These characteristics are similar to those reported for the general public in Seoul, Inchon, and Gyunggi. The mean EQ-5D index was 0.971 (standard deviation 0.08; median 1.000).

**Table 1:** Basic characteristics of the study participants

		Number	Percent
Gender	Female	182	50.6
	Male	178	49.4
Age group (years)	19–29	67	18.6
	30–39	73	20.3
	40–49	81	22.5
	50–59	70	19.4
	60-	69	19.2
Region	Seoul	148	41.1
	Incheon	41	11.4
	Gyunggi	171	47.5
Education level (years)	−8	6	1.7
	9–11	33	9.2
	12–15	224	62.2
	16-	97	26.9
		Mean (standard deviation)	
Self perceived health related quality of life (EQ-5D-3L index)		0.971 (0.08)	

Table 2 presents the means and standard deviations of the EQ-5D-3L indices according to the severity levels of 35 diseases. The raw survey data related EQ-5D-3L indices are available in the Additional file 1. The cutoff points were also calculated using the averages of the mean values of the EQ-5D-3L index for the severity levels of the health states. In the case of asthma, the EQ-5D-3L indices according to severity level were 0.956 (controlled), 0.849 (partially controlled), and 0.717 (uncontrolled). The cutoff points were 0.902 (between controlled and partially controlled) and 0.783 (between partially controlled and uncontrolled).

**Table 2:** Characteristics of the EQ-5D-3L index for diseases by severity level

No	Disease	Severity level	Response number	Mean	SD	Cut-off
1	Infectious disease	Acute episode, mild	30	0.934	0.068	-
		Acute episode, moderate	30	0.802	0.099	0.868
		Acute episode, severe	30	0.521	0.236	0.661
2	Diarrhoea	Mild	30	0.753	0.168	-
		Moderate	30	0.644	0.203	0.699
		Severe	30	0.353	0.287	0.498
3	Angina pectoris	Mild	30	0.687	0.372	-
		Moderate	30	0.663	0.318	0.675
		Severe	30	0.506	0.324	0.585
4	Heart failure	Mild	30	0.793	0.184	-
		Moderate	30	0.688	0.236	0.741
		Severe	30	0.478	0.206	0.583
5	Stroke	Long-term consequences, mild	30	0.567	0.276	-
		Long-term consequences, moderate	30	0.491	0.287	0.529
		Long-term consequences, moderate plus cognition problems	30	0.311	0.348	0.401
		Long-term consequences, severe	30	−0.035	0.197	0.138
		Long-term consequences, severe plus cognition problems	30	−0.092	0.139	−0.064
6	Asthma	Controlled	30	0.956	0.072	-
		Partially controlled	30	0.849	0.139	0.902
		Uncontrolled	30	0.717	0.228	0.783
7	COPD & other respiratory problems	Mild	30	0.929	0.108	-
		Moderate	30	0.742	0.191	0.835
		Severe	30	0.620	0.245	0.681
8	Dementia	Mild	30	0.840	0.175	-
		Moderate	30	0.648	0.223	0.744
		Severe	30	0.181	0.407	0.415
9	Multiple sclerosis	Mild	30	0.785	0.153	-
		Moderate	30	0.648	0.197	0.717
		Severe	30	0.556	0.266	0.602
10	Epilepsy	Treated, seizure free	30	0.686	0.207	-
		Treated, with recent seizure	30	0.545	0.225	0.615
		Untreated	30	0.542	0.258	0.544
		Severe	30	0.345	0.303	0.443
11	Parkinson’s disease	Mild	30	0.849	0.127	-
		Moderate	30	0.686	0.191	0.767
		Severe	30	0.344	0.333	0.515
12	Alcohol use disorder	Mild	30	0.755	0.220	-
		Moderate	30	0.730	0.207	0.743
		Severe	30	0.494	0.247	0.612
13	Fetal alcohol syndrome	Mild	30	0.850	0.136	-
		Moderate	30	0.731	0.135	0.79
		Severe	30	0.426	0.256	0.579
14	Anxiety disorder	Mild	30	0.927	0.080	-
		Moderate	30	0.835	0.117	0.881
		Severe	30	0.612	0.282	0.723
15	Major depressive disorder	Mild	30	0.813	0.157	-
		Moderate	30	0.436	0.345	0.624
		Severe	30	0.159	0.366	0.298
16	Intellectual disability	Mild	30	0.662	0.311	-
		Moderate	30	0.685	0.215	0.673
		Severe	30	0.444	0.291	0.564
		Profound	30	0.383	0.286	0.414
17	Hearing loss	Mild	30	0.877	0.167	-
		Moderate	30	0.720	0.204	0.799
		Severe	30	0.710	0.170	0.715
		Profound	30	0.528	0.206	0.619
		Complete	30	0.366	0.312	0.447
18	Hearing loss with ringing	Mild	30	0.675	0.319	-
		Moderate	30	0.635	0.248	0.655
		Severe	30	0.528	0.317	0.581
		Profound	30	0.454	0.282	0.491
		Complete	30	0.368	0.287	0.411
19	Distant vision	Mild impairment	30	0.949	0.109	-
		Moderate impairment	30	0.719	0.199	0.834
		Severe impairment	30	0.429	0.333	0.574
		Blindness	30	0.221	0.296	0.325
20	Low back pain	Acute without leg pain	30	0.446	0.363	-
		Acute with leg pain	30	0.308	0.357	0.377
		Chronic without leg pain	30	0.342	0.351	0.325
		Chronic with leg pain	30	0.188	0.272	0.265
21	Neck pain	Acute mild	30	0.694	0.169	-
		Acute severe	30	0.504	0.28	0.599
		Chronic mild	30	0.544	0.209	0.524
		Chronic severe	30	0.361	0.323	0.453
22	Musculoskeletal problems: leg	Mild	30	0.786	0.068	-
		Moderate	30	0.726	0.057	0.756
		Severe	30	0.539	0.185	0.633
23	Musculoskeletal problems: arms	Mild	30	0.438	0.354	-
		Moderate	30	0.317	0.320	0.377
24	Musculoskeletal problems: generalised	Moderate	30	0.360	0.314	-
		Severe	30	0.111	0.307	0.235
25	Abdominopelvic problem	Mild	30	0.882	0.063	-
		Moderate	30	0.699	0.156	0.791
		Severe	30	0.238	0.250	0.469
26	Disfigurement	Level 1	30	0.866	0.093	-
		Level 2	30	0.736	0.186	0.801
		Level 3	30	0.662	0.26	0.699
27	Disfigurement: with itch or pain	Level 1	30	0.721	0.183	-
		Level 2	30	0.551	0.255	0.636
		Level 3	30	0.145	0.272	0.348
28	Motor impairment	Mild	30	0.817	0.151	-
		Moderate	30	0.648	0.151	0.733
		Severe	30	0.129	0.330	0.389
29	Motor plus cognitive impairment	Mild	30	0.622	0.236	-
		Moderate	30	0.394	0.335	0.508
		Severe	30	−0.004	0.237	0.195
30	Traumatic brain injury	long-term consequences, minor with or without treatment	30	0.513	0.254	-
		long-term consequences, moderate with or without treatment	30	0.161	0.279	0.337
		long-term consequences, severe with or without treatment	30	−0.001	0.319	0.080
31	Anemia	Mild	60	0.802	0.287	-
		Moderate	60	0.596	0.313	0.699
		Severe	60	0.416	0.335	0.506
32	Allergic rhinitis and conjunctivitis	Mild	30	0.694	0.288	-
		Moderate	30	0.645	0.269	0.670
33	Annoyance	Mild	30	0.805	0.264	-
		Severe	30	0.676	0.305	0.740
34	Sleep disturbance	Mild	30	0.894	0.111	-
		Severe	30	0.807	0.208	0.851
35	Cognitive impairment in children	Mild	30	0.838	0.258	-
		Severe	30	0.816	0.181	0.827

*SD* standard deviation, *COPD* chronic obstructive pulmonary disease

Some health states had negative mean values for their EQ-5D-3L indices. For example, the mean values of the EQ-5D-3L indices for “stroke: long-term consequences, severe” and “stroke: long-term consequences, severe plus cognition problems” were −0.035 and −0.092, respectively. Consequently, the cutoff point between “stroke: long-term consequences, severe” and “stroke: long-term consequences, severe plus cognition problems” was also negative at −0.064. However, the other cutoff values were all positive.

The severity distributions for 8 diseases were estimated using these cutoff values: asthma, angina, stroke, chronic obstructive pulmonary disease (COPD), major depressive disorder, musculoskeletal problem in legs, anemia, and allergic rhinitis and conjunctivitis (Table 3). The severity distributions of the other diseases, such as dementia and epilepsy, could not be estimated because the participants who had these diseases (such as dementia or epilepsy) did not have an EQ −5D profile in both KNHNES and KCHS. Overall, the proportion of participants with mild disease severity was larger than the proportion of moderate or severe disease severity for each disease. For example, the proportions of “stroke: long-term consequences, mild” were 86.4 % (KNHNES) and 81.0 % (KCHS), whereas those of “stroke: long-term consequences, severe” were only 1.9 % (KNHNES) and 5.0 % (KCHS). In the case of major depressive disorder, the distributions of severity were 88.8 % (mild), 9.8 % (moderate), and 1.5 % (severe) according to KNHNES. However, the proportions of severe cases with asthma, COPD, and musculoskeletal problems in the legs were >10 %. In particular, the severity distributions for asthma were 52.4 % (controlled), 14.4 % (partially controlled), and 33.2 % (uncontrolled) according to KCHS.

**Table 3:** Estimated disease severity distributions

No	Disease	Severity level	KNHNES		KCHS	
			%	Year	%	Year
3	Angina pectoris	Mild	87.6	2005–2012	88.2	2008–2012
		Moderate	3.1		2.1	
		Severe	9.3		9.7	
5	Stroke	Long-term consequences, mild	86.4	2005–2012	81.0	2008–2012
		Long-term consequences, moderate	4.9		5.4	
		Long-term consequences, moderate plus cognition problems	6.5		6.0	
		Long-term consequences, severe	1.9		5.0	
		Long-term consequences, severe plus cognition problems	0.2		2.6	
6	Asthma	Controlled	53.9	2005–2012	52.4	2008–2012
		Partially controlled	17.9		14.4	
		Uncontrolled	28.2		33.2	
7	COPD & other respiratory problems	Mild	66.5	2005–2012		
		Moderate	23.3			
		Severe	10.1			
15	Major depressive disorder	Mild	88.8	2007–2012	86.1	2009–2012
		Moderate	9.8		10.8	
		Severe	1.5		3.1	
20	Low back pain	Acute without leg pain			97.7	2008
		Acute with leg pain			0.2	
		Chronic without leg pain			0.3	
		Chronic with leg pain			1.7	
22	Musculoskeletal problems: leg	Mild	74.5	2005–2012	71.2	2008
		Moderate	14.3		17.0	
		Severe	11.2		11.7	
31	Anemia	Mild	91.9	2005–2009	90.4	2008,2012
		Moderate	6.1		6.9	
		Severe	2.1		2.7	
32	Allergic rhinitis and conjunctivitis	Mild	97.9	2005–2009	98.0	2008–2012
		Moderate	2.1		2.0	

*KNHNES* Korean National Health and Nutrition Examination Surveys, *KCHS* Korean Community Health Survey, *COPD* chronic obstructive pulmonary disease

## Discussion

We have estimated the severity distributions of 8 diseases (asthma, angina, stroke, COPD, major depressive disorder, musculoskeletal problem in legs, anemia, and allergic rhinitis and conjunctivitis) using EQ-5D-3L. We performed face-to-face interviews, in which the survey participants completed the EQ-5D-3L for a hypothetical person as depicted by the lay descriptions explaining the health states of diseases. The EQ-5D-3L index was calculated for each health state using survey data obtained by this study, and the cutoff points for the severity distributions of each disease were determined according to the averages of the means of the EQ-5D-3L index for the severity levels of the health states. These cutoff points were applied to disease prevalence data obtained from population surveys performed at the national level (KNHNES and KCHS), and the severity distributions for each disease were estimated.

In terms of methodology, this study approach is similar to the indirect elicitation methods used to generate HRQoL weights [14]. The generic preference-based instruments such as EQ-5D and Health Utilities Index are generally used to evaluate status of health states developed to cover key aspects including physical and mental health in the indirect elicitation method. Although the measured aspects of health will differ depending on the instrument, it is easy to perform similar studies and comparability can be assured across diseases and countries. If there are prevalence data about HRQoL in other countries, it will be worth conducting similar studies in situations that lack data on disease severity distributions.

There is a paucity of data on disease severity distributions, although data on prevalence are relatively accessible [7]. Even though data on severity distributions are available, generalizability is limited in terms of the study designs used to collect data [15–17] and evaluate disease severity [18]. If there are national survey data on severity distributions in a certain country [19], the applicability of that data to other countries will be restricted due to differences in race, socio-demographics, and healthcare system accessibility. When collecting epidemiologic data, including prevalence and incidence, data on severity distributions are also needed to fundamentally solve this problem.

In our present study, we used 2 different population survey data sets (KNHNES and KCHS) to estimate the severity distributions. The estimated patterns for severity distribution using KNHNES and KCHS were quite similar. For example, in the case of angina pectoris, the severity distributions according to KNHNES were 87.6 % (mild), 3.1 % (moderate), and 9.3 % (severe). The severity distributions according to KCHS were 88.2 % (mild), 2.1 % (moderate), and 9.7 % (severe). These consistent results between the 2 population surveys data indicate that the reliability of this study is fair.

Overall, the proportion of participants with mild disease severity tended to be larger than moderate or severe disease severity for each disease included in this study. Because KNHNES and KCHS surveyed the general public, there is a possibility that the proportions of moderate or severe disease were underestimated. When compared with the results of other epidemiologic studies, some studies show similar results, whereas other studies demonstrate divergent results. For example, Lee et al reported that 51.8 % of their participants were stage 1 on the BODE index (reflecting the systemic nature of COPD), followed by 24.3 % at stage 2, 16.3 % at stage 3, and 7.6 % at stage 4 [18]. In this study, we estimated the severity distributions of COPD as follows: 66.5 % (mild), 23.3 % (moderate), and 10.1 % (severe). Furthermore, Cho et al suggested that the majority of individuals with low-back pain demonstrate low-intensity or disabling pain [17]. In this study, we also estimated that the proportion of cases with complicated, low-back pain was small.

According to a multinational survey on asthma, however, only 27 % of patients from South Korea reported having asthma that was well or completely controlled [20]. In our present study, we predicted that 53.9 and 52.4 % of people with asthma were in control of their disease according to KNHNES and KCHS data, respectively. These results could be due to limitations in the EQ-5D-3L used to evaluate asthma HRQoL. That is, EQ-5D-3L might not reflect all aspects of asthma, so further studies that use similar methods as this study, including disease-specific HRQoL instruments, will be needed to verify the reasons for the gap between reports.

This study has several limitations. First, we estimated the EQ-5D-3L indices and cutoff points for 35 diseases by severity, but the severity distributions were only determined for 8 diseases due to limitations in the population survey data. In KNHNES and KCHS, there are no prevalence-based data for undetermined diseases such as Parkinson’s diseases or sleep disturbance. However, if prevalence-based data with HRQoL are generated, we would be able to estimate the severity distributions of other diseases using the cutoff points from our analyses. Second, the survey participants were asked to complete EQ-5D-3L for hypothetical persons in the order of good health states. If our participants had completed the EQ-5D-3L for hypothetical people in the order of bad health states, different EQ-5D-3L indices might have been estimated. Third, when applying the cut-off points from the survey to the EQ-5D-3L indices of the KNHES and KCHS, we could not consider comorbidity in the KNHES and KCHS due to the limitation of data source. A person with a certain disease may have other diseases in the KNHES and KCHS, therefore, reported EQ-5D-3L indices in a certain disease may be influenced by concomitant diseases. Comparing a person without any comorbidity in a certain disease, the reported EQ-5D-3L indices in a certain disease would be underestimated and the proportions of severe cases would be overestimated.

## Conclusions

Using EQ-5D-3L, our present study has provided the severity distributions of 8 diseases (asthma, angina, stroke, COPD, major depressive disorder, musculoskeletal problem in legs, anemia, and allergic rhinitis and conjunctivitis) in the Korean population. Using our approach, valid disease burden could be calculated in the future in South Korea and other countries using disease severity distributions.

## Additional file

Additional file 1: Availability of data and materials. (XLSX 204 kb)

## Abbreviations

COPD: chronic obstructive pulmonary disease
DALYs: disability adjusted life years
GBD: global burden of disease
HRQoL: health related quality of life
KCHS: Korean Community Health Survey
KNHNES: Korean National Health and Nutrition Examination Surveys
YLDs: years lived with disability
YLLs: years of life lost
